# Synthesis and crystal structure of ABW-type SrFe_1.40_V_0.60_O_4_

**DOI:** 10.1107/S205698902000496X

**Published:** 2020-04-17

**Authors:** Thomas Gstir, Volker Kahlenberg, Hannes Krüger, Simon Penner

**Affiliations:** a University of Innsbruck, Institute of Mineralogy & Petrography, Innrain 52, A-6020 Innsbruck, Austria; b University of Innsbruck, Department of Physical Chemistry, Innrain 52c, A-6020 Innsbruck, Austria

**Keywords:** crystal structure, cation substitution, solid solution, topology, zeolite, ABW

## Abstract

Single crystals of SrFe_1.40_V_0.60_O_4_, strontium tetra­oxidodi[ferrate(III)/vanad­ate(III)], have been obtained as a side product in the course of sinter experiments aimed at the synthesis of double perovskites in the system SrO–Fe_2_O_3_–V_2_O_5_. The crystal structure can be characterized by layers of six-membered rings of *T*O_4_-tetra­hedra (*T*: Fe^III^, V^III^) perpendicular to [100]. Stacking of the layers along [100] results in a three-dimensional framework enclosing tunnel-like cavities in which Sr^II^ cations are incorporated for charge compensation. The sequence of directedness of up (*U*) and down (*D*) pointing vertices of neighboring tetra­hedra in a single six-membered ring is *UUUDDD*. The topology of the tetra­hedral framework belongs to the zeolite-type ABW.

## Abstract 2

A member of the novel solid-solution series SrFe_*x*_V_2–*x*_O_4_ (*x* = 1.40) has been structurally characterized. Topologically, the compound belongs to the zeolite-type ABW.

## Chemical context   

Solid oxide fuel cell (SOFC) technology is considered as particularly promising for energy storage applications (Larminie *et al.*, 2003 ▸). SOFCs are electrochemical devices that consist of three main parts: (i) a redox-capable porous cathode that reduces O_2_ to O^2–^ anions, (ii) an electrolyte transporting these anions to the anode, and (iii) the anode, where the fuel (hydrogen or carbon-containing fuels) is electro-oxidized by the O^2–^ anions to CO_2_ and H_2_O (Huang & Goodenough, 2009 ▸). Double perovskites with the general composition *A*_2_(*BB*′)O_6_ have been studied intensively as potential anode materials in SOFCs (Xu *et al.*, 2019 ▸). In the course of an explorative study on double perovskites combining mixed ionic-electronic conductivity with catalytic activity for fuel oxidation, we tried to synthesize Sr_2_FeVO_6_ using a ceramic synthesis route in the range between 1473 and 1573 K. For the highest reaction temperature, where partial melting occurred, a member of the previously unknown SrFe_*x*_V_2–*x*_O_4_ solid-solution series was observed as a side-product, and the crystal structure of the member with *x* = 1.40 is reported here.

## Structural commentary   

**Figure 1:**
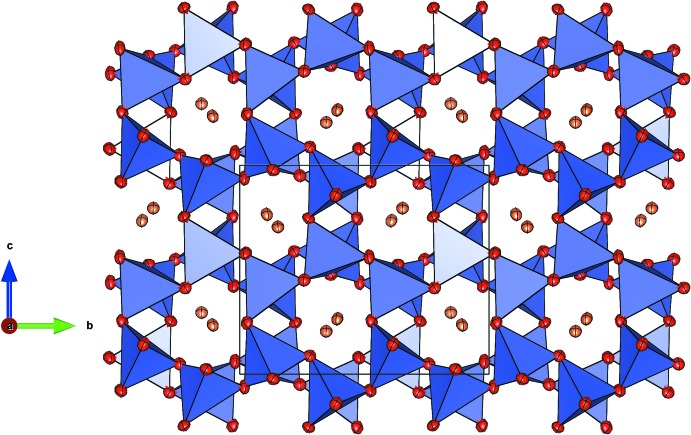
Projection of the framework structure along [100]. [*T*O_4_] tetra­hedra are shown in blue. Oxygen and strontium atoms are given in red and orange, respectively. Displacement ellipsoids are drawn at the 70% probability level.

SrFe_1.40_V_0.60_O_4_ exhibits a three-dimensional framework of corner-linked *T*O_4_-tetra­hedra (*T*: Fe^III^, V^III^). Charge compensation is achieved by the incorporation of Sr^II^ cations residing in tunnel-like cavities running parallel to [100] (Fig. 1 ▸). The compound is isostructural with SrFe_2_O_4_ (Kahlenberg & Fischer, 2001 ▸) and γ-SrGa_2_O_4_ (Kahlenberg *et al.*, 2000 ▸).

All atoms occupy general positions. Fe <—> V substitutions occur on each of the four symmetrically non-equivalent *T*-sites occupying the centers of distorted tetra­hedra formed by oxygen atoms. Site-population refinements indicate no clear trend when comparing the individual Fe:V distributions. The Fe:V population at the *T*-sites is more or less balanced ranging from 64 (3) to 75 (3)% of iron. Individual *T–*-O distances adopt values between 1.820 (6) and 1.901 (5) Å. The distortion of the tetra­hedra is also reflected in the variation of the O—*T*—O bond angles scattering between 98.2 (2) and 129.9 (2)°. According to Robinson *et al.* (1971 ▸), the distortions can be expressed numerically by means of the quadratic elongation *λ* and the angle variance *σ* ^2^. These two parameters exhibit values between 1.009 and 1.016 for λ and 34.72 and 59.96 for σ^2^.

**Figure 2:**
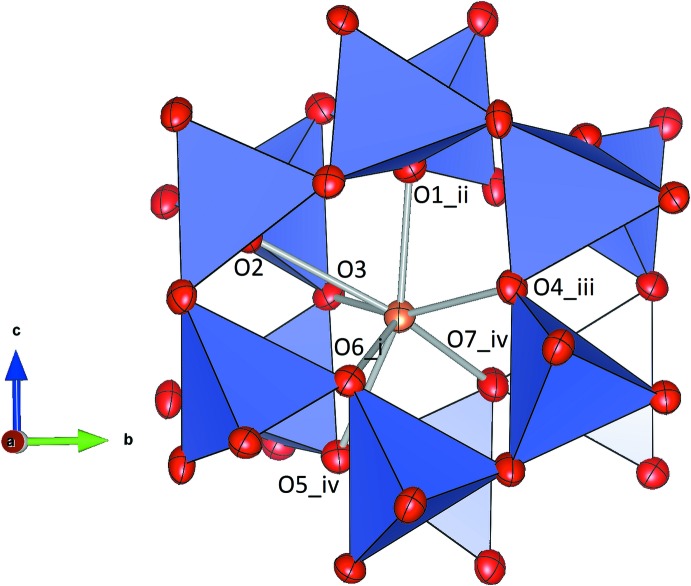
Representation of the coordination polyhedron around Sr1. Displacement ellipsoids are drawn at the 70% probability level. [Symmetry codes: (i) 1 − *x*, 1 − *y*, 1 − *z*; (ii) 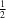 + *x*, 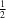 − *y*, −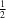 + *z*; (iii) 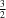 − *x*, 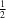 + *y*, 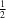 − *z*; (iv) −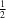 + *x*, 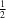 − *y*, −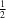 + *z*].

Each of the two symmetrically independent Sr^II^ cations is coordinated by seven oxygen atoms within the channels of the framework. They are located off-center and have irregular coordination spheres formed by the oxygen atoms of two adjacent six-membered tetra­hedral rings (Figs. 2 ▸, 3 ▸). Bond-valence-sum calculations using the parameter sets for the Sr—O bonds given by Brown & Altermatt (1985 ▸) resulted in the following values (in v.u.) considering cation–anion inter­actions up to 3.2 Å: Sr1: 1.911 and Sr2: 1.692. The considerable underbonding of the Sr2 position indicates that the bonds are stretched and that this Sr site resides in a cavity that is too large. A similar situation has been observed in isostructural SrFe_2_O_4_ and *γ*-SrGa_2_O_4_.

## Topological features   

**Figure 4:**
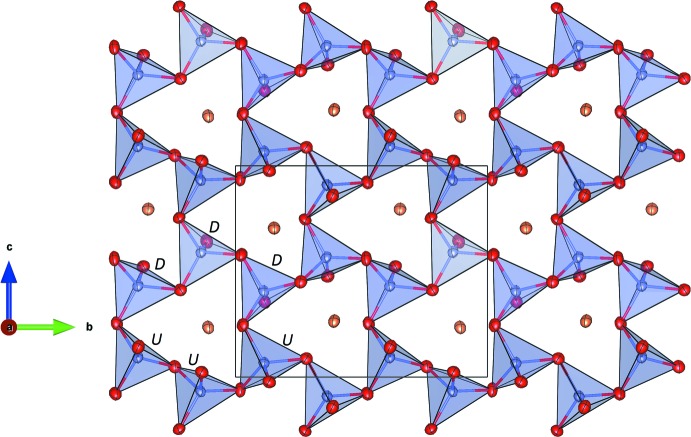
Single tetra­hedral layer with six-membered rings in a projection along [100]. *T*-sites in the centres of the tetra­hedra are shown in blue. Displacement ellipsoids are drawn at the 70% probability level.

**Figure 5:**
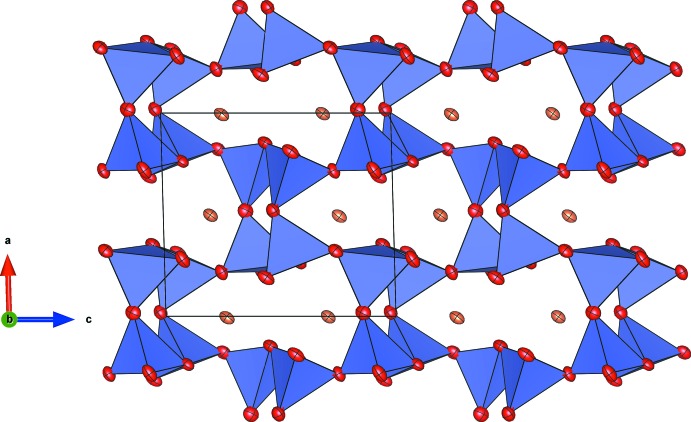
Strongly folded tetra­hedral layer with four- and eight-membered rings in a projection along [010]. Displacement ellipsoids are drawn at the 70% probability level.

SrFe_1.40_V_0.60_O_4_ belongs to the ABW zeolite structure type (Baerlocher *et al.*, 2007 ▸). This class of materials comprises a large number of representatives and has been investigated in great detail because of the complex phase transitions and inter­esting ferroic effects (Bu *et al.*, 1997 ▸). The polyhedral connectivity results in a three-dimensional network built from six-, four- and eight-membered rings. Perpendicular to [100], for example, the structure can be decomposed into layers consisting of six-membered rings (S6R) of [*T*O_4_]-tetra­hedra forming honeycomb nets (Fig. 4 ▸). Within a single S6R, three tetra­hedra with vertices up (*U*) alternate with three tetra­hedra having their vertices down (*D*) (sequence of directedness: *UUUDDD*). Using the terminology of Flörke (1967 ▸), the relative orientation of paired tetra­hedra belonging to different adjacent layers can be approximately classified as a *trans*-configuration (Fig. 1 ▸). Alternatively, the layers can be regarded as being constructed from the condensation of unbranched *vierer* single-chains *via* common corners. Perpendicular to [010] the network contains strongly corrugated layers of S4R and S8R (Fig. 5 ▸). The S8Rs are highly elliptical. Subsequent layers are connected by bridging vertex oxygen atoms, forming eight-ring channels that propagate along [010]. The elliptical shape of the channels is also reflected in the high framework density (Brunner & Meyer, 1989 ▸), with a value of 20.0 tetra­hedral atoms/1000 Å^3^.

## Synthesis and initial characterization   

Single-crystals of SrFe_1.40_V_0.60_O_4_ were obtained in the course of a series of synthesis experiments aimed at the preparation of a possible double perovskite phase with composition Sr_2_FeVO_6_. Therefore, mixtures of the dried starting materials SrCO_3_, Fe_2_O_3_ and V_2_O_5_ were homogenized in the molar ratio 4:1:1 using a ball mill operated at 600 r.p.m. for 45 min under ethanol. The resulting slurry was dried for 24 h at 323 K and subsequently re-ground by hand. An amount of about 0.5 g was pressed into a pellet having a diameter of 12 mm. Thermal treatment was performed in a resistance-heated horizontal tube furnace in air. Therefore, the tablet was placed on a platinum foil contained in an alumina-ceramic combustion boat. The sample was heated from 298 K to 1473 K with a ramp of 100 K h^−1^, followed by 25 K h^−1^ to 1423 K and finally at 10 h K^−1^ to 1573 K. After annealing for 48 h at the maximum temperature, the container was quenched to room temperature. The partially melted pellet was removed from the foil, crushed in an agate mortar and transferred to a glass slide under a reflected-light microscope. A first optical inspection revealed the presence of at least two different crystalline phases: (*a*) larger, transparent–colorless crystals up to 150 µm in size and (*b*) considerably smaller, opaque black–brown specimens with maximum dimensions of about 50 µm. Preliminary single-crystal diffraction experiments revealed the larger crystals to be Sr_3_(VO_4_)_2_ (Carrillo-Cabrera & von Schnering, 1993 ▸) while the second phase could be indexed with a monoclinic primitive unit cell similar to the one reported for SrFe_2_O_4_ (Kahlenberg & Fischer, 2001 ▸). Since the larger samples of the second phase always exhibited inter­growth of several crystals, we finally decided to focus on the fraction with smaller crystallites and to perform the relevant diffraction studies for structure elucidation using synchrotron radiation at the X06DA beamline of the Swiss Light Source, Paul Scherrer Institute, Villigen, Switzerland. Therefore, a sample was mounted on the tip of a 0.25 mm diameter LithoLoop made by Mol­ecular Dimensions Inc. with a drop of Paratone-N oil (Hampton Research) and flash cooled in a 100 K nitro­gen gas stream.

## Refinement   

Crystal data, data collection and structure refinement details are summarized in Table 1 ▸. Initial coordinates for the refinement calculations were taken from the crystal structure refinement of SrFe_2_O_4_ (Kahlenberg & Fischer, 2001 ▸) after transformation to monoclinic second setting. Site-population refinements of the Fe:V ratios on the *T*-sites indicated the presence of a member of the solid-solution series SrFe_*x*_V_2–*x*_O_4_.

## Supplementary Material

Crystal structure: contains datablock(s) global, I. DOI: 10.1107/S205698902000496X/wm5552sup1.cif

Structure factors: contains datablock(s) I. DOI: 10.1107/S205698902000496X/wm5552Isup2.hkl

CCDC reference: 1995764

Additional supporting information:  crystallographic information; 3D view; checkCIF report

## Figures and Tables

**Figure 3:**
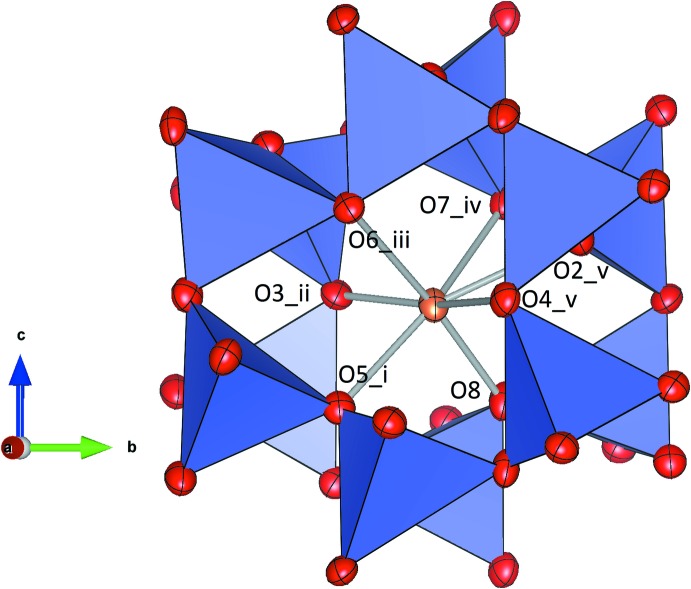
Representation of the coordination polyhedron around Sr2. Ellipsoids are drawn at the 70% level. [Symmetry codes: (i) 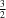 − *x*, 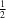 + *y*, 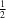 − *z*; (ii) 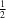 − *x*, 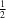 + *y*, 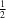 − *z*; (iii) 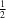 + *x*, 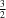 − *y*, −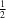 + *z*; (iv) 1 − *x*, 1 − *y*, 1 − *z*; (v) *x*, 1 + *y*, *z*]

**Table 1:** Experimental details

Crystal data	
Chemical formula	SrFe_1.40_V_0.60_O_4_
*M*_r_	260.37
Crystal system, space group	Monoclinic, *P*2_1_/*n*
Temperature (K)	100
*a*, *b*, *c* (Å)	8.0594 (8), 10.8768 (9), 9.1218 (8)
β (°)	91.544 (7)
*V* (Å^3^)	799.33 (12)
*Z*	8
Radiation type	Synchrotron, λ = 0.72931 Å
μ (mm^−1^)	20.91
Crystal size (mm)	0.03 × 0.02 × 0.01

Data collection	
Diffractometer	Aerotech
Absorption correction	Multi-scan (*CrysAlis PRO*; Rigaku OD, 2018 ▸)
*T*_min_, *T*_max_	0.614, 0.871
No. of measured, independent and observed [*I* > 2σ(*I*)] reflections	5200, 1746, 1572
*R*_int_	0.067
(sin θ/λ)_max_ (Å^−1^)	0.641

Refinement	
*R*[*F* ^2^ > 2σ(*F* ^2^)], *wR*(*F* ^2^), *S*	0.050, 0.144, 1.14
No. of reflections	1746
No. of parameters	131
Δρ_max_, Δρ_min_ (e Å^−3^)	1.74, −1.37

Computer programs: *CrysAlis PRO* (Rigaku OD, 2018 ▸), *SHELXL97* (Sheldrick, 2008 ▸), *VESTA* (Momma & Izumi, 2011 ▸), *publCIF* (Westrip, 2010 ▸) and *WinGX* (Farrugia, 2012 ▸).

## supplementary crystallographic information

### Crystal data

**Table:**

SrFe_1.40_V_0.60_O_4_	*F*(000) = 961.6
*M**_r_* = 260.37	*D*_x_ = 4.327 Mg m^−^^3^
Monoclinic, *P*2_1_/*n*	Synchrotron radiation, λ = 0.72931 Å
Hall symbol: -P 2yn	Cell parameters from 2398 reflections
*a* = 8.0594 (8) Å	θ = 3.5–33.9°
*b* = 10.8768 (9) Å	µ = 20.91 mm^−^^1^
*c* = 9.1218 (8) Å	*T* = 100 K
β = 91.544 (7)°	Fragment, brown-black
*V* = 799.33 (12) Å^3^	0.03 × 0.02 × 0.01 mm
*Z* = 8	

### Data collection

**Table:**

Aerotech diffractometer	1746 independent reflections
Radiation source: SLS super-bending magnet 2.9T, X06DA	1572 reflections with *I* > 2σ(*I*)
Bartels Monochromator with dual channel cut crystals (DCCM) in (±∓) geometry monochromator	*R*_int_ = 0.067
Detector resolution: 5.81 pixels mm^-1^	θ_max_ = 27.9°, θ_min_ = 3.0°
rotation method scans	*h* = −9→10
Absorption correction: multi-scan (*CrysAlis PRO*; Rigaku OD, 2018)	*k* = −13→13
*T*_min_ = 0.614, *T*_max_ = 0.871	*l* = −11→11
5200 measured reflections	

### Refinement

**Table:**

Refinement on *F*^2^	0 restraints
Least-squares matrix: full	Primary atom site location: isomorphous structure methods
*R*[*F*^2^ > 2σ(*F*^2^)] = 0.050	Secondary atom site location: notdet
*wR*(*F*^2^) = 0.144	*w* = 1/[σ^2^(*F*_o_^2^) + (0.0821*P*)^2^] where *P* = (*F*_o_^2^ + 2*F*_c_^2^)/3
*S* = 1.14	(Δ/σ)_max_ < 0.001
1746 reflections	Δρ_max_ = 1.74 e Å^−^^3^
131 parameters	Δρ_min_ = −1.37 e Å^−^^3^

### Special details

**Table:**

Geometry. All esds (except the esd in the dihedral angle between two l.s. planes) are estimated using the full covariance matrix. The cell esds are taken into account individually in the estimation of esds in distances, angles and torsion angles; correlations between esds in cell parameters are only used when they are defined by crystal symmetry. An approximate (isotropic) treatment of cell esds is used for estimating esds involving l.s. planes.
Refinement. Refinement of F^2^ against ALL reflections. The weighted R-factor wR and goodness of fit S are based on F^2^, conventional R-factors R are based on F, with F set to zero for negative F^2^. The threshold expression of F^2^ > 2σ(F^2^) is used only for calculating R-factors(gt) etc. and is not relevant to the choice of reflections for refinement. R-factors based on F^2^ are statistically about twice as large as those based on F, and R- factors based on ALL data will be even larger.

### Fractional atomic coordinates and isotropic or equivalent isotropic displacement parameters (Å^2^)

**Table:**

	*x*	*y*	*z*	*U*_iso_*/*U*_eq_	Occ. (<1)
Sr1	0.49713 (9)	0.34684 (7)	0.20513 (7)	0.0190 (3)	
Sr2	0.50716 (9)	0.89165 (6)	0.23511 (8)	0.0193 (3)	
Fe1	0.22326 (15)	0.13896 (10)	0.06706 (12)	0.0163 (4)	0.64 (3)
Fe2	0.29893 (14)	0.13638 (10)	0.40844 (12)	0.0164 (4)	0.75 (3)
Fe3	0.17887 (15)	0.88717 (9)	0.93230 (12)	0.0166 (4)	0.71 (4)
Fe4	0.74301 (15)	0.11238 (9)	0.40681 (12)	0.0159 (4)	0.70 (3)
V1	0.22326 (15)	0.13896 (10)	0.06706 (12)	0.0163 (4)	0.36 (3)
V2	0.29893 (14)	0.13638 (10)	0.40844 (12)	0.0164 (4)	0.25 (3)
V3	0.17887 (15)	0.88717 (9)	0.93230 (12)	0.0166 (4)	0.29 (4)
V4	0.74301 (15)	0.11238 (9)	0.40681 (12)	0.0159 (4)	0.30 (3)
O1	0.0192 (7)	0.1337 (5)	0.9770 (6)	0.0218 (11)	
O2	0.5181 (7)	0.1144 (5)	0.3576 (6)	0.0235 (12)	
O3	0.2158 (6)	0.2238 (5)	0.2460 (5)	0.0208 (11)	
O4	0.8217 (6)	0.0290 (5)	0.2457 (5)	0.0209 (11)	
O5	0.8530 (6)	0.2590 (5)	0.4510 (5)	0.0196 (10)	
O6	0.2367 (6)	0.7187 (5)	0.9126 (5)	0.0193 (11)	
O7	0.7805 (7)	0.0230 (5)	0.5784 (5)	0.0238 (12)	
O8	0.3105 (6)	0.9810 (5)	0.0607 (6)	0.0217 (11)	

### Atomic displacement parameters (Å^2^)

**Table:**

	*U*^11^	*U*^22^	*U*^33^	*U*^12^	*U*^13^	*U*^23^
Sr1	0.0171 (4)	0.0186 (4)	0.0210 (4)	−0.0003 (3)	−0.0052 (3)	0.0001 (2)
Sr2	0.0184 (5)	0.0167 (4)	0.0226 (4)	0.0007 (2)	−0.0058 (3)	−0.0016 (2)
Fe1	0.0162 (7)	0.0141 (6)	0.0182 (6)	−0.0007 (4)	−0.0042 (4)	−0.0008 (4)
Fe2	0.0161 (7)	0.0146 (6)	0.0183 (6)	0.0005 (4)	−0.0038 (4)	0.0007 (4)
Fe3	0.0163 (7)	0.0148 (7)	0.0184 (6)	−0.0006 (4)	−0.0051 (4)	−0.0002 (4)
Fe4	0.0154 (7)	0.0140 (6)	0.0182 (6)	−0.0002 (4)	−0.0047 (4)	−0.0005 (4)
V1	0.0162 (7)	0.0141 (6)	0.0182 (6)	−0.0007 (4)	−0.0042 (4)	−0.0008 (4)
V2	0.0161 (7)	0.0146 (6)	0.0183 (6)	0.0005 (4)	−0.0038 (4)	0.0007 (4)
V3	0.0163 (7)	0.0148 (7)	0.0184 (6)	−0.0006 (4)	−0.0051 (4)	−0.0002 (4)
V4	0.0154 (7)	0.0140 (6)	0.0182 (6)	−0.0002 (4)	−0.0047 (4)	−0.0005 (4)
O1	0.024 (3)	0.022 (3)	0.019 (3)	−0.003 (2)	−0.002 (2)	−0.001 (2)
O2	0.024 (3)	0.020 (3)	0.027 (3)	0.002 (2)	−0.002 (2)	−0.004 (2)
O3	0.025 (3)	0.018 (3)	0.019 (2)	0.001 (2)	−0.0046 (19)	0.0019 (19)
O4	0.022 (3)	0.018 (3)	0.023 (2)	0.005 (2)	−0.003 (2)	0.002 (2)
O5	0.018 (3)	0.019 (2)	0.022 (2)	−0.001 (2)	−0.0044 (18)	0.001 (2)
O6	0.016 (3)	0.020 (3)	0.021 (2)	−0.002 (2)	−0.0041 (18)	0.0004 (19)
O7	0.031 (3)	0.018 (3)	0.022 (3)	−0.002 (2)	−0.009 (2)	0.0020 (19)
O8	0.021 (3)	0.015 (3)	0.028 (3)	0.000 (2)	−0.009 (2)	0.001 (2)

### Geometric parameters (Å, º)

**Table:**

Sr1—O1^i^	2.490 (5)	Fe4—O5	1.863 (5)
Sr1—O4^ii^	2.495 (5)	O1—V1^xiii^	1.820 (6)
Sr1—O7^iii^	2.506 (5)	O1—Fe1^xiii^	1.820 (6)
Sr1—O6^iv^	2.527 (5)	O1—V3^xii^	1.832 (6)
Sr1—O3	2.668 (5)	O1—Fe3^xii^	1.832 (6)
Sr1—O5^iii^	2.811 (5)	O1—Sr1^xiv^	2.490 (5)
Sr1—O2	2.889 (5)	O2—Sr2^ix^	2.668 (5)
Sr2—O8	2.419 (5)	O3—Sr2^xv^	2.571 (5)
Sr2—O5^ii^	2.517 (5)	O4—V3^iv^	1.862 (5)
Sr2—O3^v^	2.571 (5)	O4—Fe3^iv^	1.862 (5)
Sr2—O2^vi^	2.668 (5)	O4—Sr1^xvi^	2.495 (5)
Sr2—O6^vii^	2.706 (5)	O4—Sr2^ix^	2.942 (5)
Sr2—O4^vi^	2.942 (5)	O5—V1^xvii^	1.872 (5)
Sr2—O7^iv^	3.057 (6)	O5—Fe1^xvii^	1.872 (5)
Fe1—O1^viii^	1.820 (6)	O5—Sr2^xvi^	2.517 (5)
Fe1—O8^ix^	1.858 (5)	O5—Sr1^xvii^	2.811 (5)
Fe1—O5^iii^	1.872 (5)	O6—V2^xviii^	1.891 (5)
Fe1—O3	1.877 (5)	O6—Fe2^xviii^	1.891 (5)
Fe2—O7^x^	1.853 (6)	O6—Sr1^iv^	2.527 (5)
Fe2—O2	1.854 (6)	O6—Sr2^xix^	2.706 (5)
Fe2—O3	1.869 (5)	O7—V2^x^	1.853 (6)
Fe2—O6^xi^	1.891 (5)	O7—Fe2^x^	1.853 (6)
Fe3—O1^xii^	1.832 (6)	O7—Sr1^xvii^	2.506 (5)
Fe3—O4^iv^	1.862 (5)	O7—Sr2^iv^	3.057 (6)
Fe3—O8^xiii^	1.863 (5)	O8—V1^vi^	1.858 (5)
Fe3—O6	1.901 (5)	O8—Fe1^vi^	1.858 (5)
Fe4—O4	1.853 (5)	O8—V3^viii^	1.863 (5)
Fe4—O2	1.855 (6)	O8—Fe3^viii^	1.863 (5)
Fe4—O7	1.860 (5)		
			
O1^i^—Sr1—O4^ii^	74.21 (17)	V1^xiii^—O1—Fe3^xii^	126.0 (3)
O1^i^—Sr1—O7^iii^	116.19 (18)	Fe1^xiii^—O1—Fe3^xii^	126.0 (3)
O4^ii^—Sr1—O7^iii^	91.79 (17)	V3^xii^—O1—Fe3^xii^	0.00 (7)
O1^i^—Sr1—O6^iv^	114.11 (17)	V1^xiii^—O1—Sr1^xiv^	119.1 (3)
O4^ii^—Sr1—O6^iv^	78.52 (16)	Fe1^xiii^—O1—Sr1^xiv^	119.1 (3)
O7^iii^—Sr1—O6^iv^	123.51 (17)	V3^xii^—O1—Sr1^xiv^	114.9 (3)
O1^i^—Sr1—O3	86.65 (17)	Fe3^xii^—O1—Sr1^xiv^	114.9 (3)
O4^ii^—Sr1—O3	150.33 (16)	Fe2—O2—Fe4	150.7 (3)
O7^iii^—Sr1—O3	76.34 (16)	Fe2—O2—Sr2^ix^	101.6 (2)
O6^iv^—Sr1—O3	130.71 (16)	Fe4—O2—Sr2^ix^	96.4 (2)
O1^i^—Sr1—O5^iii^	150.77 (17)	Fe2—O2—Sr1	87.9 (2)
O4^ii^—Sr1—O5^iii^	134.45 (15)	Fe4—O2—Sr1	99.8 (2)
O7^iii^—Sr1—O5^iii^	65.40 (15)	Sr2^ix^—O2—Sr1	126.3 (2)
O6^iv^—Sr1—O5^iii^	82.57 (15)	Fe2—O3—Fe1	114.8 (3)
O3—Sr1—O5^iii^	64.90 (14)	Fe2—O3—Sr2^xv^	123.0 (2)
O1^i^—Sr1—O2	66.00 (15)	Fe1—O3—Sr2^xv^	116.5 (2)
O4^ii^—Sr1—O2	125.61 (16)	Fe2—O3—Sr1	94.5 (2)
O7^iii^—Sr1—O2	138.40 (18)	Fe1—O3—Sr1	94.5 (2)
O6^iv^—Sr1—O2	85.32 (17)	Sr2^xv^—O3—Sr1	104.54 (18)
O3—Sr1—O2	62.13 (16)	Fe4—O4—V3^iv^	117.3 (3)
O5^iii^—Sr1—O2	93.22 (15)	Fe4—O4—Fe3^iv^	117.3 (3)
O8—Sr2—O5^ii^	94.82 (17)	V3^iv^—O4—Fe3^iv^	0.00 (6)
O8—Sr2—O3^v^	83.26 (16)	Fe4—O4—Sr1^xvi^	117.2 (2)
O5^ii^—Sr2—O3^v^	87.97 (16)	V3^iv^—O4—Sr1^xvi^	122.2 (2)
O8—Sr2—O2^vi^	85.67 (18)	Fe3^iv^—O4—Sr1^xvi^	122.2 (2)
O5^ii^—Sr2—O2^vi^	142.41 (18)	Fe4—O4—Sr2^ix^	87.81 (19)
O3^v^—Sr2—O2^vi^	129.21 (18)	V3^iv^—O4—Sr2^ix^	103.8 (2)
O8—Sr2—O6^vii^	175.50 (17)	Fe3^iv^—O4—Sr2^ix^	103.8 (2)
O5^ii^—Sr2—O6^vii^	80.68 (15)	Sr1^xvi^—O4—Sr2^ix^	95.86 (17)
O3^v^—Sr2—O6^vii^	96.47 (15)	Fe4—O5—V1^xvii^	111.1 (3)
O2^vi^—Sr2—O6^vii^	97.91 (16)	Fe4—O5—Fe1^xvii^	111.1 (3)
O8—Sr2—O4^vi^	111.49 (16)	V1^xvii^—O5—Fe1^xvii^	0.00 (8)
O5^ii^—Sr2—O4^vi^	84.97 (15)	Fe4—O5—Sr2^xvi^	124.3 (2)
O3^v^—Sr2—O4^vi^	164.10 (15)	V1^xvii^—O5—Sr2^xvi^	108.0 (2)
O2^vi^—Sr2—O4^vi^	60.41 (16)	Fe1^xvii^—O5—Sr2^xvi^	108.0 (2)
O6^vii^—Sr2—O4^vi^	68.34 (14)	Fe4—O5—Sr1^xvii^	90.66 (18)
O8—Sr2—O7^iv^	75.56 (16)	V1^xvii^—O5—Sr1^xvii^	90.07 (17)
O5^ii^—Sr2—O7^iv^	155.43 (15)	Fe1^xvii^—O5—Sr1^xvii^	90.07 (17)
O3^v^—Sr2—O7^iv^	68.68 (15)	Sr2^xvi^—O5—Sr1^xvii^	127.46 (19)
O2^vi^—Sr2—O7^iv^	60.55 (17)	V2^xviii^—O6—Fe2^xviii^	0.00 (9)
O6^vii^—Sr2—O7^iv^	108.56 (14)	V2^xviii^—O6—Fe3	109.4 (2)
O4^vi^—Sr2—O7^iv^	119.55 (14)	Fe2^xviii^—O6—Fe3	109.4 (2)
O1^viii^—Fe1—O8^ix^	107.2 (2)	V2^xviii^—O6—Sr1^iv^	112.6 (2)
O1^viii^—Fe1—O5^iii^	106.0 (2)	Fe2^xviii^—O6—Sr1^iv^	112.6 (2)
O8^ix^—Fe1—O5^iii^	108.2 (2)	Fe3—O6—Sr1^iv^	121.7 (2)
O1^viii^—Fe1—O3	111.0 (2)	V2^xviii^—O6—Sr2^xix^	100.9 (2)
O8^ix^—Fe1—O3	120.2 (2)	Fe2^xviii^—O6—Sr2^xix^	100.9 (2)
O5^iii^—Fe1—O3	103.4 (2)	Fe3—O6—Sr2^xix^	108.6 (2)
O7^x^—Fe2—O2	103.2 (2)	Sr1^iv^—O6—Sr2^xix^	101.25 (16)
O7^x^—Fe2—O3	114.2 (2)	V2^x^—O7—Fe2^x^	0.00 (7)
O2—Fe2—O3	101.0 (2)	V2^x^—O7—Fe4	119.6 (3)
O7^x^—Fe2—O6^xi^	109.0 (2)	Fe2^x^—O7—Fe4	119.6 (3)
O2—Fe2—O6^xi^	116.4 (2)	V2^x^—O7—Sr1^xvii^	137.4 (2)
O3—Fe2—O6^xi^	112.6 (2)	Fe2^x^—O7—Sr1^xvii^	137.4 (2)
O1^xii^—Fe3—O4^iv^	118.2 (2)	Fe4—O7—Sr1^xvii^	100.9 (2)
O1^xii^—Fe3—O8^xiii^	105.8 (2)	V2^x^—O7—Sr2^iv^	88.8 (2)
O4^iv^—Fe3—O8^xiii^	105.6 (2)	Fe2^x^—O7—Sr2^iv^	88.8 (2)
O1^xii^—Fe3—O6	98.2 (2)	Fe4—O7—Sr2^iv^	101.6 (2)
O4^iv^—Fe3—O6	112.6 (2)	Sr1^xvii^—O7—Sr2^iv^	95.81 (17)
O8^xiii^—Fe3—O6	116.8 (2)	V1^vi^—O8—Fe1^vi^	0.00 (10)
O4—Fe4—O2	99.6 (2)	V1^vi^—O8—V3^viii^	108.4 (2)
O4—Fe4—O7	111.1 (2)	Fe1^vi^—O8—V3^viii^	108.4 (2)
O2—Fe4—O7	110.2 (3)	V1^vi^—O8—Fe3^viii^	108.4 (2)
O4—Fe4—O5	114.8 (2)	Fe1^vi^—O8—Fe3^viii^	108.4 (2)
O2—Fe4—O5	119.9 (2)	V3^viii^—O8—Fe3^viii^	0.00 (6)
O7—Fe4—O5	101.5 (2)	V1^vi^—O8—Sr2	126.4 (2)
V1^xiii^—O1—Fe1^xiii^	0.00 (6)	Fe1^vi^—O8—Sr2	126.4 (2)
V1^xiii^—O1—V3^xii^	126.0 (3)	V3^viii^—O8—Sr2	123.1 (2)
Fe1^xiii^—O1—V3^xii^	126.0 (3)	Fe3^viii^—O8—Sr2	123.1 (2)

Symmetry codes: (i) *x*+1/2, −*y*+1/2, *z*−1/2; (ii) −*x*+3/2, *y*+1/2, −*z*+1/2; (iii) *x*−1/2, −*y*+1/2, *z*−1/2; (iv) −*x*+1, −*y*+1, −*z*+1; (v) −*x*+1/2, *y*+1/2, −*z*+1/2; (vi) *x*, *y*+1, *z*; (vii) *x*+1/2, −*y*+3/2, *z*−1/2; (viii) *x*, *y*, *z*−1; (ix) *x*, *y*−1, *z*; (x) −*x*+1, −*y*, −*z*+1; (xi) −*x*+1/2, *y*−1/2, −*z*+3/2; (xii) −*x*, −*y*+1, −*z*+2; (xiii) *x*, *y*, *z*+1; (xiv) *x*−1/2, −*y*+1/2, *z*+1/2; (xv) −*x*+1/2, *y*−1/2, −*z*+1/2; (xvi) −*x*+3/2, *y*−1/2, −*z*+1/2; (xvii) *x*+1/2, −*y*+1/2, *z*+1/2; (xviii) −*x*+1/2, *y*+1/2, −*z*+3/2; (xix) *x*−1/2, −*y*+3/2, *z*+1/2.
